# Dengue death with evidence of hemophagocytic syndrome and dengue virus infection in the bone marrow

**DOI:** 10.1186/s40064-015-1463-z

**Published:** 2015-11-02

**Authors:** Hasliana Azrah Ab-Rahman, Pooi-Fong Wong, Hafiz Rahim, Juraina Abd-Jamil, Kim-Kee Tan, Syuhaida Sulaiman, Chai-See Lum, Syarifah-Faridah Syed-Omar, Sazaly AbuBakar

**Affiliations:** Tropical Infectious Diseases Research and Education Centre (TIDREC), Faculty of Medicine, University of Malaya, 50603 Kuala Lumpur, Malaysia; Department of Medical Microbiology, Faculty of Medicine, University of Malaya, 50603 Kuala Lumpur, Malaysia; Department of Pharmacology, Faculty of Medicine, University of Malaya, 50603 Kuala Lumpur, Malaysia; Department of Medicine, Faculty of Medicine, University of Malaya, 50603 Kuala Lumpur, Malaysia; Department of Paediatrics, Faculty of Medicine, University of Malaya, 50603 Kuala Lumpur, Malaysia

**Keywords:** Dengue, Hemophagocytic syndrome, Bone marrow, Ferritin, Macrophage, MAS

## Abstract

**Introduction:**

HPS is a potentially life-threatening histiocytic disorder that has been described in various viral infections including dengue. Its involvement in severe and fatal dengue is probably more common but is presently under recognized.

**Case description:**

A 38-year-old female was admitted after 5 days of fever. She was deeply jaundiced, leukopenic and thrombocytopenic. Marked elevation of transaminases, hyperbilirubinemia and hypoalbuminemia were observed. She had deranged INR values and prolonged aPTT accompanied with hypofibrinogenemia. She also had splenomegaly. She was positive for dengue IgM. Five days later she became polyuric and CT brain image showed gross generalized cerebral edema. Her conditions deteriorated by day 9, became confused with GCS of 9/15. Her BMAT showed minimal histiocytes. Her serum ferritin level peaked at 13,670.00 µg/mL and her sCD163 and sCD25 values were markedly elevated at 4750.00 ng/mL and 4191.00 pg/mL, respectively. She succumbed to the disease on day 10 and examination of her tissues showed the presence of dengue virus genome in the bone marrow.

**Discussion and evaluation:**

It is described here, a case of fatal dengue with clinical features of HPS. Though BMAT results did not show the presence of macrophage hemophagocytosis, other laboratory features were consistent with HPS especially marked elevation of ferritin, sCD163 and sCD25. Detection of dengue virus in the patient’s bone marrow, fifteen days after the onset of fever was also consistent with the suggestion that the HPS is associated with dengue virus infection.

**Conclusions:**

The findings highlight HPS as a possible complication leading to severe dengue and revealed persistent dengue virus infection of the bone marrow. Detection of HPS markers; ferritin, sCD163 and sCD25, therefore, should be considered for early recognition of HPS-associated dengue.

## Background

Dengue is recognized as one of the most important vector-borne human diseases (World Health Organization and the Special Programme for Research and Training in Tropical Diseases (TDR) 2009). The disease is endemic in many tropical and subtropical regions of the world (World Health Organization and the Special Programme for Research and Training in Tropical Diseases (TDR) 2009; Brady et al. 2012). Dengue usually presents as mild dengue fever and most patients will recover without complications. Less than 2 % of the dengue patients would present with the severe forms of the disease, dengue hemorrhagic fever (DHF) and dengue shock syndrome (DSS) which are characterized by severe intravascular leakages that could lead to hypovolemic shock and multi-organ failures (Avirutnam et al. 2006; Wills et al. 2004; Martina et al. 2009). Dengue deaths usually occur within this group of dengue patients (Sam et al. 2013; Campos et al. 2015). While dengue is still a pediatric health concern, the infection has become more of a young adult disease in many endemic regions (Alexander et al. 2011). A number of clinical presentations of dengue, which are typical to adult infections, have been documented and this has become the impetus for World Health Organization (WHO) to revise its original classification of dengue to take into account of other ‘atypical’ manifestations of dengue (Rowe et al. 2014; Nimmagadda et al. 2014; Hadinegoro 2012). Under the recent WHO classification, most of the dengue patients fall under the group of dengue with warning signs (Alexander et al. 2011) which include abdominal pain or tenderness, persistent vomiting, mucosal bleeding, hepatomegaly, lethargy or restlessness, thrombocytopenia and increased hematocrit profile. Severe dengue is often reported with co-morbidities such as diabetes mellitus, obesity, hypertension and asthma (Thein et al. 2013; Zakaria et al. 2014; Saqib et al. 2014). In addition to these, other atypical manifestations including encephalitis, rhabdomyolysis, acute motor quadriparesis, and hemophagocytic syndrome (HPS) have also been described for severe dengue (Araujo et al. 2012; Mok et al. 2013; Gutch et al. 2012; Ramanathan and Duraisamy 1991; Rueda et al. 2002; Lu et al. 2005; Nakamura et al. 2009; Soler et al. 2010; Tan et al. 2012; Sharp et al. 2014; Riberio et al. 2014; De Koninck et al. 2014; Mitra and Bhattacharya 2014; Pal et al. 2014; Phuakpet et al. 2015).

HPS is characterized by uncontrolled activation of normal T lymphocytes and macrophages, leading to overwhelming production of pro-inflammatory cytokines which can cause hyperinflammation (Ravelli et al. 2012). HPS-associated dengue disease was reported as early as 1966 (Janka 2012), and to date at least 44 case reports of HPS in dengue have been reported in the literature. HPS has been described especially in severe dengue (Ramanathan and Duraisamy 1991; Rueda et al. 2002; Lu et al. 2005; Nakamura et al. 2009; Soler et al. 2010; Tan et al. 2012; Sharp et al. 2014; Riberio et al. 2014; De Koninck et al. 2014; Mitra and Bhattacharya 2014; Pal et al. 2014; Phuakpet et al. 2015) and reports of HPS in confirmed dengue cases, presented clinical and diagnostic features similar to those of HPS which include hyperferritinemia and hemophagocytosis activity in the bone marrow. However, most of these earlier reports did not include the evidence of other distinct features of HPS such as elevated levels of soluble CD163 (sCD163) and soluble CD25 (sCD25) (Ravelli et al. 2012; Bleesing et al. 2007). Even though the number of reported cases of HPS in dengue in the literature is low, it is possible that the incidence could be much higher due to its under-recognition. Here we report a case of fatal dengue with typical HPS presentations including the detection of dengue virus in the bone marrow and the use of serum biomarkers of HPS in the detection of severe dengue.

## Case report

A 38-year-old female was admitted to the University of Malaya Medical Centre (UMMC), Kuala Lumpur after 5 days of fever with right hypochondriac region abdominal pain, persistent vomiting, myalgia, headache and an episode of epistaxis. She also complained of passing tea-colored urine for 3 days duration and 2 days history of dry cough with right-sided pleuritic chest pain. There was no recent travel history and other household members were well. She denied any substance abuse, had blood transfusion or consumed any herbal or traditional medicines. There was no past medical and family history of connective tissue diseases (CTD) or hepatitis. She was not on any immunosuppressant or other regular medications and CTD screening was not performed. On admission, she was alert and deeply jaundiced. She was found to be febrile (38.6 °C), normotensive (110/70 mmHg) but tachycardic (100 bpm). There was no flapping tremor to suggest hepatic encephalopathy. The abdomen was generally soft with tender hepatomegaly. Cardiovascular, respiratory and neurological examinations were unremarkable.

**Fig. 1:**
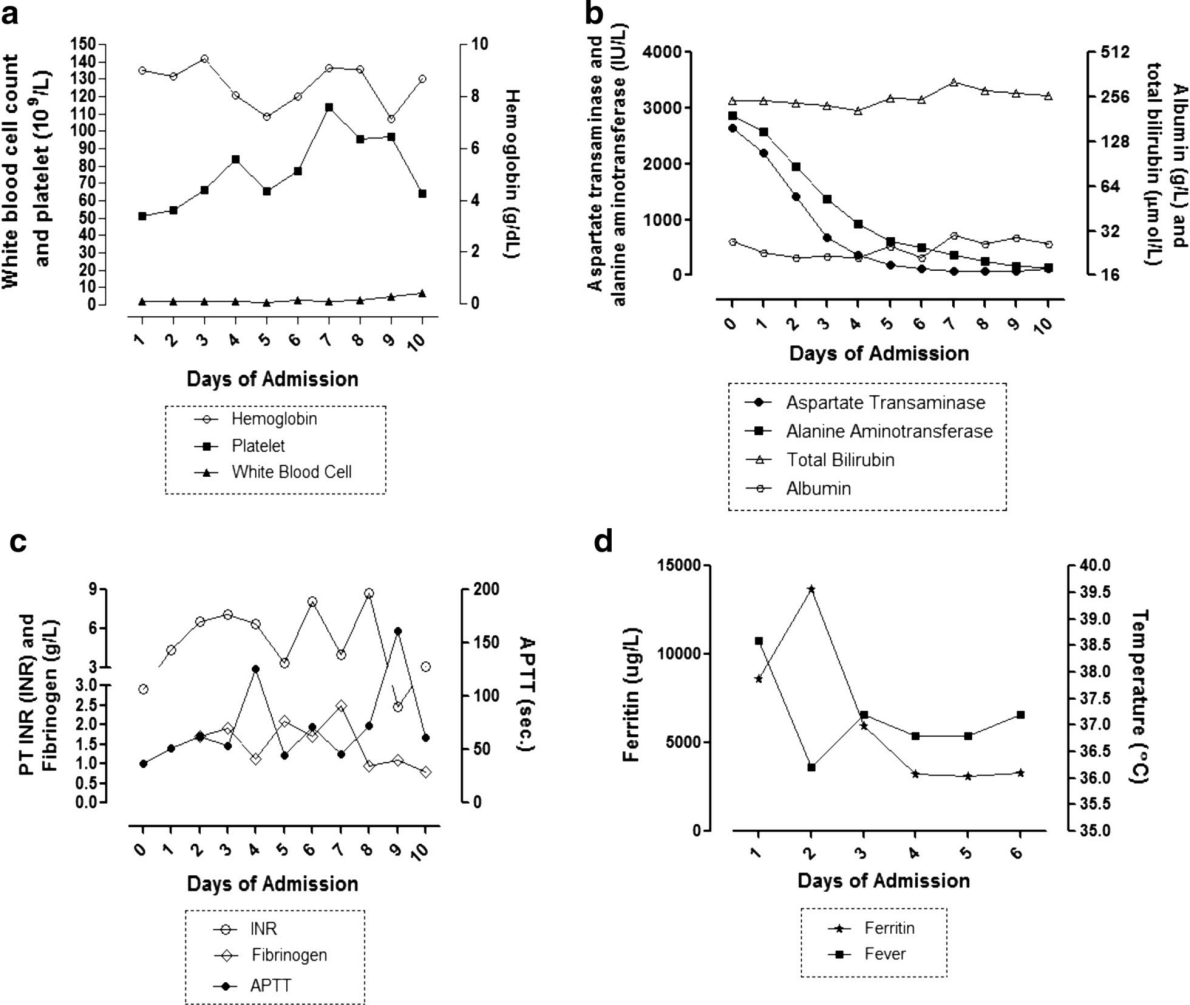
Laboratory diagnostic profiles of a dengue patient with HPS presentation. **a** Full blood count analysis **b** Liver function tests **c** Coagulation profile **d** Body temperature and level of ferritin. *INR* International normalised ratio, *aPTT* activated partial thromboplastin time

Her full blood count showed leukopenia and thrombocytopenia (Fig. 1a). Her liver function tests showed marked elevation of transaminases as well as hyperbilirubinemia and hypoalbuminemia (Fig. 1b). Her coagulation profile showed deranged international normalized ratio (INR) values and prolonged activated partial thromboplastin time (aPTT) accompanied with hypofibrinogenemia (Fig. 1c). Abdominal ultrasound revealed non-specific increased in peri-portal echogenicity with ‘starry sky’ appearance, consistent with acute hepatitis. There was neither biliary obstruction nor ascites noted. She was hydrated with maintenance normal saline drip of 1 cc/kg/h. She was empirically started on intravenous infusion (IVI) Ceftriaxone and given intravenous vitamin K 10 mg once daily. She was tested negative for malaria, hepatitis B and C. Paracetamol and salicylate levels were undetectable. Blood cultures were negative for any bacterial or fungal growth.

At day 2 of admission (day 7 of illness), she remained alert but tachycardic. IgM serology test for dengue was positive. Clinical examination revealed minimal right-sided pleural effusion. In view of the worsening coagulation profile, she was transfused with 4 units of fresh frozen plasma and four units of cryoprecipitate. There was no bleeding tendency. Throughout her stay, her urine output and blood pressure were satisfactory. Thrombotic thrombocytopenic purpura (TTP) was ruled out as the peripheral blood film (PBF) was not suggestive and her renal function was normal. However, she was found to have splenomegaly.

By day 4 of hospitalization (day 9 of illness), she became confused and drowsy. Her Glasgow Coma Scale (GCS) was 9/15 (E3V1M5). Kernig’s sign was negative. Pupils were 5 mm equal and reactive bilaterally. She remained afebrile. Her blood pressure (BP) was 105/75 mmHg with heart rate 144 beats per minute. There was no focal neurological deficit with bilateral down-going plantar. Plain computed tomography (CT) of the brain showed no evidence of intracranial bleed or focal lesion. In view of the profound thrombocytopenia as well as marked coagulopathy, a lumbar puncture was not performed. She was electively intubated due to deteriorating conscious level and for airway protection. Broad-spectrum antibiotics were introduced which included IVI meropenem and IVI azithromycin. Blood products were transfused in an attempt to correct her coagulopathy. The gastroenterology team was consulted for her worsening liver function and she was started on IVI N-acetyl cysteine. Her serum ferritin on day 6 of illness was 6307.33 µg/L, suggesting possible macrophage activation syndrome (MAS) or HPS secondary to dengue virus infection. Intravenous dexamethasone of 8.00 mg TID was started. A bone marrow trephine and aspiration (BMAT) was performed to confirm this diagnosis.

On day 5 of hospitalization (day 10 of illness), she became polyuric. A repeated CT brain imaging showed gross generalized cerebral oedema. Further investigations confirmed a diagnosis of cranial diabetes insipidus (DI). The hematologist reviewed the BMAT, which showed minimal histiocytes that was less likely to be diagnostic for HLH. However, a diagnosis of dengue virus infection associated with subsequent HPS was established due to elevated serum level of HPS biomarkers and other clinical diagnostic criteria (Pal et al. 2014). Serum HPS biomarkers including ferritin, haptoglobin, soluble CD163 (sCD163) and soluble CD25 (sCD25) were measured. Her ferritin level peaked on day 2 of hospitalization (day 7 of illness) at 13,670.00 µg/mL and decreased thereafter (Fig. 1d). Soluble haptoglobin, CD163 and CD25 determined using commercially available enzyme-linked immunosorbent assay kits (Genway, San Diego; R&D System, Minneapolis) showed a very low level of haptoglobin (0.05 g/L) compared to healthy donor group with mean value of 228.49 g/L (data not shown). Highly elevated levels of sCD163 and sCD25 with values of 4750.00 ng/mL and 4191.00 pg/mL, respectively, in comparison to mean values of 261.65 ng/mL (sCD163) and 483.69 pg/mL (sCD25) of a group of healthy donors (n = 39) (data not shown) were observed. Despite our best effort and aggressive medical intervention, the patient succumbed to her ordeal by day 10 of hospitalization.

Tissue specimens of bone marrow, peritoneal fluid and throat swab obtained during post-mortem were used for nucleic acid amplification assays using general Flavivirus amplification primers (Teoh et al. 2013). Only the bone marrow tissue sample was positive for partial sequences of non-structural proteins NS1, NS2A, NS4b and NS5. Bidirectional DNA sequencing results confirmed the presence of nucleotide sequences matching that of DENV-2 with 99 % sequence similarity (data not shown).

Dengue viruses can cause a spectrum of disease ranging from mild flu-like fever to hemorrhagic fever with severe intravascular leakages and hypovolemic shock (Martina et al. 2009). The 2009 revised World Health Organization (WHO) dengue classification characterized severe dengue as dengue with severe plasma leakage, severe organ impairment or severe hemorrhage (World Health Organization and the Special Programme for Research and Training in Tropical Diseases (TDR) 2009). Although a clear molecular mechanism leading to severe dengue is still not well understood, it is thought that macrophage activation syndrome may have a role in some cases of severe dengue (Ramanathan and Duraisamy 1991; Rueda et al. 2002; Lu et al. 2005; Nakamura et al. 2009; Soler et al. 2010; Tan et al. 2012; Sharp et al. 2014; Riberio et al. 2014; De Koninck et al. 2014; Mitra and Bhattacharya 2014; Pal et al. 2014; Phuakpet et al. 2015). The increasing reports of hemophagocytosis associated with dengue infection in both endemic and non-endemic areas have raised concern of the potential threat of this emerging syndrome in causing severe and fatal complications of dengue (Ramanathan and Duraisamy 1991; Rueda et al. 2002; Lu et al. 2005; Nakamura et al. 2009; Soler et al. 2010; Tan et al. 2012; Sharp et al. 2014; Riberio et al. 2014; De Koninck et al. 2014; Mitra and Bhattacharya 2014; Pal et al. 2014; Phuakpet et al. 2015).

Clinically, according to the 2009 revised World Health Organization (WHO) dengue classification, the patient in our study met the criteria of severe dengue with presentations of severe hemorrhage and liver failure. Her low haptoglobin level provided evidence of bleeding and liver impairment, resulting in poor coagulation profile and markedly elevated liver enzymes. Furthermore, our initial suspicion that the patient was having HPS as a possible complication was supported by laboratory findings of elevated levels of serum ferritin, sCD163 and sCD25 typical of HPS (Ravelli et al. 2012; Bleesing et al. 2007). Although observation of hemophagocytosis is the gold standard for identifying macrophage activation syndrome, specific biomarkers are needed for its early detection. In this case, serum ferritin, sCD163 and sCD25 levels were charted during the course of her hospitalization. Elevated transaminases levels were also observed; and this was similar to an earlier observation which supports the possibility of HPS contributing to hepatic dysfunction (Lu et al. 2005). We also detected dengue viral RNA in the bone marrow tissue samples, providing direct evidence of the presence of dengue virus in the tissue. This was consistent with previous reports highlighting the involvement of bone marrow in dengue virus infection (Nelson et al. 1966; Nisalak et al. 1970). It is also worth noting that the dengue virus persisted in the bone marrow even at 15 days post onset of fever and long after viremia was over. Currently, it is not known how long dengue virus would persist in the marrow following an infection.

## Conclusions

We present here a fatal case of dengue with clinical and laboratory findings suggesting the involvement of HPS and evidence supporting dengue virus infection of the bone marrow, detectable even on day 15 after the onset of fever. These findings emphasized HPS as a serious complication of dengue and that dengue virus persisted in the bone marrow long after viremia is over. A reliable treatment strategy for combating dengue-associated HPS, however, is yet to be established. Early detection of HPS biomarkers and treatment with methylprednisolone or dexamethasone at appropriate dosage may prevent further complications and death (Mitra and Bhattacharya 2014; Wan Jamaluddin et al. 2015) but this requires further investigation.

## Consent

A written informed consent was obtained. The study received approval from the University of Malaya Medical Centre (UMMC) Medical Ethics Committee (Ethics Number—908.9). The study conformed to the Declaration of Helsinki and Malaysian Good Clinical Practice (GCP) guidelines.

## Abbreviations

HPS: hemophagocytic syndrome
WHO: World Health Organization
DHF: dengue hemorrhagic fever
DSS: dengue shock syndrome
UMMC: University of Malaya Medical Centre
CTD: connective tissue diseases
INR: international normalized ratio
aPTT: activated partial thromboplastin time
IVI: intravenous infusion
TTP: thrombotic thrombocytopenic purpura
PBF: peripheral blood film
GSC: glasgow coma scale
BP: blood pressure
CT: computed tomography
MAS: macrophage activation syndrome
TID: ter in die
DI: diabetes insipidus
HLH: hemophagocytic lymphohistiocytosis
sCD163: soluble CD163
sCD25: soluble CD25
